# Patients’ Experiences With Digitalization in the Health Care System: Qualitative Interview Study

**DOI:** 10.2196/47278

**Published:** 2024-04-11

**Authors:** Christian Gybel Jensen, Frederik Gybel Jensen, Mia Ingerslev Loft

**Affiliations:** 1 Department of Neurology Rigshospitalet Copenhagen Denmark; 2 Institute for People and Technology Roskilde University Roskilde Denmark

**Keywords:** digitalization, digital health, eHealth, digital health literacy, digital practices, patient experiences, digital health services, inequity in health, qualitative research, interview, implementation, tool, neurology, digital tool, communication, mobile phone

## Abstract

**Background:**

The digitalization of public and health sectors worldwide is fundamentally changing health systems. With the implementation of digital health services in health institutions, a focus on digital health literacy and the use of digital health services have become more evident. In Denmark, public institutions use digital tools for different purposes, aiming to create a universal public digital sector for everyone. However, this digitalization risks reducing equity in health and further marginalizing citizens who are disadvantaged. Therefore, more knowledge is needed regarding patients’ digital practices and experiences with digital health services.

**Objective:**

This study aims to examine digital practices and experiences with public digital health services and digital tools from the perspective of patients in the neurology field and address the following research questions: (1) How do patients use digital services and digital tools? (2) How do they experience them?

**Methods:**

We used a qualitative design with a hermeneutic approach. We conducted 31 semistructured interviews with patients who were hospitalized or formerly hospitalized at the department of neurology in a hospital in Denmark. The interviews were audio recorded and subsequently transcribed. The text from each transcribed interview was analyzed using manifest content analysis.

**Results:**

The analysis provided insights into 4 different categories regarding digital practices and experiences of using digital tools and services in health care systems: social resources as a digital lifeline, possessing the necessary capabilities, big feelings as facilitators or barriers, and life without digital tools. Our findings show that digital tools were experienced differently, and specific conditions were important for the possibility of engaging in digital practices, including having access to social resources; possessing physical, cognitive, and communicative capabilities; and feeling motivated, secure, and comfortable. These prerequisites were necessary for participants to have positive experiences using digital tools in the health care system. Those who did not have these prerequisites experienced challenges and, in some cases, felt left out.

**Conclusions:**

Experiences with digital practices and digital health services are complex and multifaceted. Engagement in digital practices for the examined population requires access to continuous assistance from their social network. If patients do not meet requirements, digital health services can be experienced as exclusionary and a source of concern. Physical, cognitive, and communicative difficulties might make it impossible to use digital tools or create more challenges. To ensure that digitalization does not create inequities in health, it is necessary for developers and institutions to be aware of the differences in digital health literacy, focus on simplifying communication with patients and next of kin, and find flexible solutions for citizens who are disadvantaged.

## Introduction

### Background

In 2022, the fourth most googled question in Denmark was, “Why does MitID not work?” [1]. MitID (My ID) is a digital access tool that Danes use to enter several different private and public digital services, from bank accounts to mail from their municipality or the state. MitID is a part of many Danish citizens’ everyday lives because the public sector in Denmark is digitalized in many areas. In recent decades, digitalization has changed how governments and people interact and has demonstrated the potential to change the core functions of public sectors and delivery of public policies and services [2]. When public sectors worldwide become increasingly digitalized, this transformation extends to the public health sectors as well, and some studies argue that we are moving toward a “digital public health era” that is already impacting the health systems and will fundamentally change the future of health systems [3]. While health systems are becoming more digitalized, it is important that both patients and digitalized systems adapt to changes in accordance with each other. Digital practices of people can be understood as what people do with and through digital technologies and how people relate to technology [4]. Therefore, it is relevant to investigate digital practices and how patients perceive and experience their own use of digital tools and services, especially in relation to existing digital health services. In our study, we highlight a broad perspective on experiences with digital practices and particularly add insight into the challenges with digital practices faced by patients who have acute or chronic illness, with some of them also experiencing physical, communicative, or cognitive difficulties.

An international Organization for Economic Cooperation and Development report indicates that countries are digitalized to different extents and in different ways; however, this does not mean that countries do not share common challenges and insights into the implementation of digital services [2].

In its global Digital Government Index, Denmark is presented as one of the leading countries when it comes to public digitalization [2]. Recent statistics indicate that approximately 97% of Danish families have access to the internet at home [5]. The Danish health sector already offers many different digital services, including web-based delivery of medicine, e-consultations, patient-related outcome questionnaires, and seeking one’s own health journal or getting test results through; “Sundhed” [6] (the national health portal) and “Sundhedsjournalen” (the electronic patient record); or the apps “Medicinkortet” (the shared medication record), “Minlæge” (My Doctor, consisting of, eg, communication with the general practitioner), or “MinSP” (My Health Platform, consisting of, eg, communication with health care staff in hospitals) [6-8].

The Danish Digital Health Strategy from 2018 aims to create a coherent and user-friendly digital public sector for everyone [9], but statistics indicate that certain groups in society are not as digitalized as others. In particular, the older population uses digital services the least, with 5% of people aged 65 to 75 years and 18% of those aged 75 to 89 years having never used the internet in 2020 [5]. In parts of the literature, it has been problematized how the digitalization of the welfare state is related to the marginalization of older citizens who are socially disadvantaged [10]. However, statistics also indicate that the probability of using digital tools increases significantly as a person’s experience of using digital tools increases, regardless of their age or education level [5].

Understanding the digital practices of patients is important because they can use digital tools to engage with the health system and follow their own health course. Researching experiences with digital practices can be a way to better understand potential possibilities and barriers when patients use digital health services. With patients becoming more involved in their own health course and treatment, the importance of patients’ health literacy is being increasingly recognized [11]. The World Health Organization defines health literacy as the “achievement of a level of knowledge, personal skills and confidence to take action to improve personal and community health by changing personal lifestyles and living conditions” [12]. Furthermore, health literacy can be described as “a person’s knowledge and competencies to meet complex demands of health in modern society,*”* and it is viewed as a critical step toward patient empowerment [11,12]. In a digitalized health care system, this also includes the knowledge, capabilities, and resources that individuals require to use and benefit from eHealth services, that is, “digital health literacy (eHealth literacy)” [13]. An eHealth literacy framework created by Norgaard et al [13] identified that different aspects, for example, the ability to process information and actively engage with digital services, can be viewed as important facets of digital health literacy. This argument is supported by studies that demonstrate how patients with cognitive and communicative challenges experience barriers to the use of digital tools and require different approaches in the design of digital solutions in the health sector [14,15]. Access to digital services and digital literacy is becoming increasingly important determinants of health, as people with digital literacy and access to digital services can facilitate improvement of health and involvement in their own health course [16].

### Objectives

The need for a better understanding of eHealth literacy and patients’ capabilities to meet public digital services’ demands as well as engage in their own health calls for a deeper investigation into digital practices and the use of digital tools and services from the perspective of patients with varying digital capabilities. Important focus areas to better understand digital practices and related challenges have already been highlighted in various studies. They indicate that social support, assessment of value in digital services, and systemic assessment of digital capabilities are important in the use and implementation of digital tools, and they call for better insight into complex experiences with digital services [13,17,18]. Therefore, we aimed to examine digital practices and experiences with public digital health services and digital tools from the perspective of patients, addressing the following research questions: how do patients use digital services and digital tools, and how do they experience them?

## Methods

### Design

We aimed to investigate digital practices and experiences with digital health services and digital tools; therefore, we used a qualitative design and adopted a hermeneutic approach as the point of departure, which means including preexisting knowledge of digital practices but also providing room for new comprehension [19]. Our interpretive approach is underpinned by the philosophical hermeneutic approach by Gadamer et al [19], in which they described the interpretation process as a “hermeneutic circle,” where the researcher enters the interpretation process with an open mind and historical awareness of a phenomenon (preknowledge). We conducted semistructured interviews using an interview guide. This study followed the COREQ (Consolidated Criteria for Reporting Qualitative Research) checklist [20].

### Setting and Participants

To gain a broad understanding of experiences with public digital health services, a purposive sampling strategy was used. All 31 participants were hospitalized or formerly hospitalized patients in a large neurological department in the capital of Denmark (Table 1). We assessed whether including patients from the neurological field would give us a broad insight into the experiences of digital practices from different perspectives. The department consisted of, among others, 8 inpatient units covering, for example, acute neurology and stroke units, from which the patients were recruited. Patients admitted to a neurological department can have both acute and transient neurological diseases, such as infections in the brain, stroke, or blood clot in the brain from which they can recover completely or have persistent physical and mental difficulties, or experience chronic neurological and progressive disorders such as Parkinson disease and dementia. Some patients hospitalized in neurological care will have communicative and cognitive difficulties because of their neurological disorders. Nursing staff from the respective units helped the researchers (CGJ, FGJ, and MIL) identify patients who differed in terms of gender, age, and severity of neurological illness. Some patients (6/31, 19%) had language difficulties; however, a speech therapist assessed them as suitable participants. We excluded patients with severe cognitive difficulties and those who were not able to speak the Danish language. Including patients from the field of neurology provided an opportunity to study the experience of digital health practice from various perspectives. Hence, the sampling strategy enabled the identification and selection of information-rich participants relevant to this study [21], which is the aim of qualitative research. The participants were invited to participate by either the first (CGJ) or last author (MIL), and all invited participants (31/31, 100%) chose to participate.

All 31 participants were aged between 40 to 99 years, with an average age of 71.75 years (Table 1). Out of the 31 participants, 10 (32%) had physical disabilities or had cognitive or communicative difficulties due to sequela in relation to neurological illness or other physical conditions.

**Table 1:** Participant demographics (N=31).

Demographics			Participant, n (%)
**Gender**			
	Men	15 (48)	
	Women	16 (52)	
**Age (years)**			
	40-49	4 (13)	
	50-59	2 (6)	
	60-69	3 (10)	
	70-79	14 (45)	
	80-89	6 (19)	
	90-99	2 (6)	
**Hospitalized or formerly hospitalized in the neurology department**			
	Interviewed only during admission	20 (65)	
	Interviewed after being discharged	11 (35)	
Patients with physical, communicative, or cognitive difficulties			10 (32)

### Data Collection

The 31 patient interviews were conducted over a 2-month period between September and November 2022. Of the 31 patients, 20 (65%) were interviewed face-to-face at the hospital in their patient room upon admission and 11 (35%) were interviewed on the phone after being discharged. The interviews had a mean length of 20.48 minutes.

We developed a semistructured interview guide (Table 2). The interview questions were developed based on the research aim, findings from our preliminary covering of literature in the field presented in the Introduction section, and identified gaps that we needed to elaborate on to be able to answer our research question [22]. The semistructured interview guide was designed to support the development of a trusting relationship and ensure the relevance of the interviews’ content [22]. The questions served as a prompt for the participants and were further supported by questions such as “please tell me more” and “please elaborate” throughout the interview, both to heighten the level of detail and to verify our understanding of the issues at play. If the participant had cognitive or communicative difficulties, communication was supported using a method called Supported Communication for Adults with Aphasia [23] during the interview.

The interviews were performed by all authors (CGJ, FGJ, and MIL individually), who were skilled in conducting interviews and qualitative research. The interviewers are not part of daily clinical practice but are employed in the department of neurology from where the patients were recruited. All interviews were audio recorded and subsequently transcribed verbatim by all 3 authors individually.

**Table 2:** Interview guide.

Main questions		Supporting questions, if needed
**Background questions**		
	Please tell me a little about yourself.	What is your gender? What is your age? What is your level of education and degree? Why were you admitted to the hospital (if they were), and have you been admitted to a hospital before?
**Questions regarding digital practices**		
	Please tell me about your digital devices.	Do you own your own computer or smartphone or a similar device? How do you use these devices?
	What is your experience with using digital devices?	Can you elaborate and give examples? Would you consider computer or smartphones a part of your everyday life? If so, in which way? Have you ever used these devices for work?
	What does it mean to you, to be able/not able to use these devices?	Can you elaborate and give examples? Have your ever wished you were better at using these devices? Is using these devices something you would like to learn? (if they have not) Could you imagine using any of these devices 1 day in the future? Why or why not?
	Please tell me how you use digital services from the public sector.	For example, do you read mail digitally? Do you access your bank account on the web?
	What do you think about the use of digital devices in the public sector?	Not available
	Please tell me how you have experienced your use of the digital services in the public sector.	Can you elaborate and give examples? Did you face any challenges? How did you handle them? (if they have any) What do you think was the cause of these challenges? How did it feel when you experienced these challenges? (if they have any)
	How did you learn to use digital devices?	Can you elaborate and give examples? Have you ever had any assistance in the use of digital devices? If so, what type of assistance? What would you have done if you could not get that assistance? Have you been offered any help to overcome these challenges? Have you ever found it difficult to talk with someone about challenges in the use of digital devices? If you got the opportunity to improve your digital skills, is this something you would consider? Why or why not? Have you ever been offered courses or classes in the use of digital devices?
	How is digital practice part of your everyday life?	Have you ever experienced having trouble doing something digitally, you thought could easily do? Have you ever experienced learning or doing something on a digital device you thought you never would? Which type of emotions would you describe as connected to the use of digital devices?
**Questions regarding digital practices in the health system**		
	Please tell me how you experience the use of digital technology in the health system.	Have you heard of sundhed.dk, and if so, do you use it and for what purpose? Have you heard of MinSP (app) and, if so, do you use it and for what purpose? Do you think these services could be made more accessible or relevant to you and, if so, how?
	With what purpose do you use digital services in the health system? (if they do)	For example, video consulting, answering PRO^a^, and ordering vaccinations
	How do you communicate with institutions in the health system?	Have you ever had to respond to something from the hospital digitally? Do you use digital devices to communicate in any form with any part of the health system? Do you read results and invitations from the hospital digitally on a computer or smartphone or tablet? Do you use any other digital services in the public health system?
	How do you experience the accessibility of these digital Health services?	Do you think these services could be made more accessible or relevant to you and, if so, how? Do you have any advice for people who do not find it easy to use digital devices? (if they are experienced)

^a^PRO: patient-related outcome.

### Data Analysis

The text from each transcribed interview was analyzed using manifest content analysis, as described by Graneheim and Lundman [24]. Content analysis is a method of analyzing written, verbal, and visual communication in a systematic way [25]. Qualitative content analysis is a structured but nonlinear process that requires researchers to move back and forth between the original text and parts of the text during the analysis. Manifest analysis is the descriptive level at which the surface structure of the text central to the phenomenon and the research question is described. The analysis was conducted as a collaborative effort between the first (CGJ) and last authors (MIL); hence, in this inductive circular process, to achieve consistency in the interpretation of the text, there was continued discussion and reflection between the researchers. The transcriptions were initially read several times to gain a sense of the whole context, and we analyzed each interview. The text was initially divided into domains that reflected the lowest degree of interpretation, as a rough structure was created in which the text had a specific area in common. The structure roughly reflected the interview guide’s themes, as guided by Graneheim and Lundman [24]. Thereafter, the text was divided into meaning units, condensed into text-near descriptions, and then abstracted and labeled further with codes. The codes were categorized based on similarities and differences. During this process, we discussed the findings to reach a consensus on the content, resulting in the final 4 categories presented in this paper.

### Ethical Considerations

The interviewees received oral and written information about the study and its voluntary nature before the interviews. Written informed consent was obtained from all participants. Participants were able to opt of the study at any time. Data were anonymized and stored electronically on locked and secured servers. The Ethics Committee of the Capitol Region in Denmark was contacted before the start of the study. This study was registered and approved by the ethics committee and registered under the Danish Data Protection Agency (number P2021-839). Furthermore, the ethical principles of the Declaration of Helsinki were followed for this study.

## Results

The analysis provided insights into 4 different categories regarding digital practices and experiences of using digital tools and services in health care systems: social resources as a digital lifeline, possessing the necessary capabilities, big feelings as facilitators or barriers, and life without digital tools.

### Social Resources as a Digital Lifeline

Throughout the analysis, it became evident that access to both material and social resources was of great importance when using digital tools. Most participants already possessed and had easy access to a computer, smartphone, or tablet. The few participants who did not own the necessary digital tools told us that they did not have the skills needed to use these tools. For these participants, the lack of material resources was tied particularly to a lack of knowledge and know-how, as they expressed that they would not know where to start after buying a computer—how to set it up, connect it to the internet, and use its many systems.

However, possessing the necessary material resources did not mean that the participants possessed the knowledge and skill to use digital tools. Furthermore, access to material resources was also a question of having access to assistance when needed. Some participants who had access to a computer, smartphone, and tablet and knew how to use these tools still had to obtain help when setting up hardware, updating software, or getting a new device. These participants were confident in their own ability to use digital devices but also relied on family, friends, and neighbors in their everyday use of these tools. Certain participants were explicitly aware of their own use of social resources when expressing their thoughts on digital services in health care systems:

I think it is a blessing and a curse. I think it is both. I would say that if I did not have someone around me in my family who was almost born into the digital world, then I think I would be in trouble. But I feel sorry for those who do not have that opportunity, and I know quite a few who do not. They get upset, and it’s really frustrating.

Woman, age 82 years

The participants’ use of social resources indicates that learning skills and using digital tools are not solely individual tasks but rather continuously involve engagement with other people, particularly whenever a new unforeseen problem arises or when the participants want a deeper understanding of the tools they are using:

If tomorrow I have to get a new ipad...and it was like that when I got this one, then I had to get XXX to come and help me move stuff and he was sweet to help with all the practical stuff. I think I would have cursed a couple of times (if he hadn’t been there), but he is always helpful, but at the same time he is also pedagogic so I hope that next time he showed me something I will be able to do it.

Man, age 71 years

For some participants, obtaining assistance from a more experienced family member was experienced as an opportunity to learn, whereas for other participants, their use of public digital services was even tied directly to assistance from a spouse or family member:

My wife, she has access to mine, so if something comes up, she can just go in and read, and we can talk about it afterwards what (it is).

Man, age 85 years

The participants used social resources to navigate digital systems and understand and interpret communication from the health care system through digital devices. Another example of this was the participants who needed assistance to find, answer, and understand questionnaires from the health care department. Furthermore, social resources were viewed as a support system that made participants feel more comfortable and safer when operating digital tools. The social resources were particularly important when overcoming unforeseen and new challenges and when learning new skills related to the use of digital tools. Participants with physical, cognitive, and communicative challenges also explained how social resources were of great importance in their ability to use digital tools.

### Possessing the Necessary Capabilities

The findings indicated that possessing the desire and knowing how to use digital tools are not always enough to engage with digital services successfully. Different health issues can carry consequences for motor skills and mobility. Some of these consequences were visibly affecting how our participants interacted with digital devices, and these challenges were somewhat easy to discover. However, our participants revealed hidden challenges that posed difficulties. In some specific cases, cognitive and communicative inabilities can make it difficult to use digital tools, and this might not always be clear until the individual tries to use a device’s more complex functions. An example of this is that some participants found it easy to turn on a computer and use it to write but difficult to go through security measures on digital services or interpret and understand digital language. Remembering passwords and logging on to systems created challenges, particularly for those experiencing health issues that directly affect memory and cognitive abilities, who expressed concerns about what they were able to do through digital tools:

I think it is very challenging because I would like to use it how I used to before my stroke; (I) wish that everything (digital skills) was transferred, but it just isn’t.

Man, age 80 years

Despite these challenges, the participants demonstrated great interest in using digital tools, particularly regarding health care services and their own well-being. However, sometimes, the challenges that they experienced could not be conquered merely by motivation and good intentions. Another aspect of these challenges was the amount of extra time and energy that the participants had to spend on digital services. A patient diagnosed with Parkinson disease described how her symptoms created challenges that changed her digital practices:

Well it could for example be something like following a line in the device. And right now it is very limited what I can do with this (iPhone). Now I am almost only using it as a phone, and that is a little sad because I also like to text and stuff, but I also find that difficult (...) I think it is difficult to get an overview.

Woman, age 62 years

Some participants said that after they were discharged from the hospital, they did not use the computer anymore because it was too *difficult* and too *exhausting*, which contributed to them *giving up*. Using digital tools already demanded a certain amount of concentration and awareness, and some diseases and health conditions affected these abilities further.

### Big Feelings as Facilitators or Barriers

The findings revealed a wide range of digital practices in which digital tools were used as a communication device, as an entertainment device, and as a practical and informative tool for ordering medicine, booking consultations, asking health-related questions, or receiving email from public institutions. Despite these different digital practices, repeating patterns and arguments appeared when the participants were asked why they learned to use digital tools or wanted to improve their skills. A repeating argument was that they wanted to “follow the times,*”* or as a participant who was still not satisfied with her digital skills stated:

We should not go against the future.

Woman, age 89 years

The participants expressed a positive view of the technological developments and possibilities that digital devices offered, and they wanted to improve their knowledge and skills related to digital practice. For some participants, this was challenging, and they expressed frustration over how technological developments “moved too fast*,”* but some participants interpreted these challenges as a way to “keep their mind sharp.*”*

Another recurring pattern was that the participants expressed great interest in using digital services related to the health care system and other public institutions. The importance of being able to navigate digital services was explicitly clear when talking about finding test answers, written electronic messages, and questionnaires from the hospital or other public institutions. Keeping up with developments, communicating with public institutions, and taking an interest in their own health and well-being were described as good reasons to learn to use digital tools.

However, other aspects also affected these learning facilitators. Some participants felt alienated while using digital tools and described the practice as something related to feelings of anxiety, fear, and stupidity as well as something that demanded “a certain amount of courage.*”* Some participants felt frustrated with the digital challenges they experienced, especially when the challenges were difficult to overcome because of their physical conditions:

I get sad because of it (digital challenges) and I get very frustrated and it takes a lot of time because I have difficulty seeing when I look away from the computer and have to turn back again to find out where I was and continue there (...) It pains me that I have to use so much time on it.

Man, age 71 years

Fear of making mistakes, particularly when communicating with public institutions, for example, the health care system, was a common pattern. Another pattern was the fear of misinterpreting the sender and the need to ensure that the written electronic messages were actually from the described sender. Some participants felt that they were forced to learn about digital tools because they cared a lot about the services. Furthermore, fears of digital services replacing human interaction were a recurring concern among the participants. Despite these initial and recurring feelings, some participants learned how to navigate the digital services that they deemed relevant. Another recurring pattern in this learning process was repetition, the practice of digital skills, and consistent assistance from other people. One participant expressed the need to use the services often to remember the necessary skills:

Now I can figure it out because now I’ve had it shown 10 times. But then three months still pass... and then I think...how was it now? Then I get sweat on my forehead (feel nervous) and think; I’m not an idiot.

Woman, age 82 years

For some participants, learning how to use digital tools demanded time and patience, as challenges had to be overcome more than once because they reappeared until the use of digital tools was more automatized into their everyday lives. Using digital tools and health services was viewed as easier and less stressful when part of everyday routines.

### Life Without Digital Tools: Not a Free Choice

Even though some participants used digital tools daily, other participants expressed that it was “too late for them.” These participants did not view it as a free choice but as something they had to accept that they could not do. They wished that they could have learned it earlier in life but did not view it as a possibility in the future. Furthermore, they saw potential in digital services, including digital health care services, but they did not know exactly what services they were missing out on. Despite this lack of knowledge, they still felt sad about the position they were in. One participant expressed what she thought regarding the use of digital tools in public institutions:

Well, I feel alright about it, but it is very, very difficult for those of us who do not have it. Sometimes you can feel left out—outside of society. And when you do not have one of those (computers)...A reference is always made to w and w (www.) and then you can read on. But you cannot do that.

Woman, age 94 years

The feeling of being left out of society was consistent among the participants who did not use digital tools. To them, digital systems seemed to provide unfair treatment based on something outside of their own power. Participants who were heavily affected by their medical conditions and could not use digital services also felt left out because they saw the advantages of using digital tools. Furthermore, a participant described the feelings connected to the use of digital tools in public institutions:

It is more annoying that it does not seem to work out in my favour.

Woman, age 62 years

These statements indicated that it is possible for individuals to want to use digital tools and simultaneously find them too challenging. These participants were aware that there are consequences of not using digital tools, and that saddens them, as they feel like they are not receiving the same treatment as other people in society and the health care system.

## Discussion

### Principal Findings

The insights from our findings demonstrated that our participants had different digital practices and different experiences with digital tools and services; however, the analysis also highlighted patterns related to how digital services and tools were used. Specific conditions were important for the possibility of digital practice, including *having access to social resources; possessing the necessary capabilities;* and *feeling motivated, secure, and comfortable*. These prerequisites were necessary to have positive experiences using digital tools in the health care system, although some participants who lived up to these prerequisites were still skeptical toward digital solutions. Others who did not live up to these prerequisites experienced challenges and even though they were aware of opportunities, this awareness made them feel left out. A few participants even viewed the digital tools as a threat to their participation in society. This supports the notion of Norgaard et al [13] that the attention paid to digital capability demands from eHealth systems is very important. Furthermore, our findings supported the argument of Hjeltholt and Papazu [17] that it is important to better understand experiences related to digital services. In our study, we accommodate this request and bring forth a broad perspective on experiences with digital practices; we particularly add insight into the challenges with digital practices for patients who also have acute or chronic illness, with some of them also experiencing physical, communicative, and cognitive difficulties. To our knowledge, there is limited existing literature focusing on digital practices that do not have a limited scope, for example, a focus on perspectives on eHealth literacy in the use of apps [26] or intervention studies with a focus on experiences with digital solutions, for example, telemedicine during the COVID-19 pandemic [27]. As mentioned by Hjeltholt et al [10], certain citizens are dependent on their own social networks in the process of using and learning digital tools. Rasi et al [28] and Airola et al [29] argued that digital health literacy is situated and should include the capabilities of the individual’s social network. Our findings support these arguments that access to social resources is an important condition; however, the findings also highlight that these resources can be particularly crucial in the use of digital health services, for example, when interpreting and understanding digital and written electronic messages related to one’s own health course or when dealing with physical, cognitive, and communicative disadvantages. Therefore, we argue that the awareness of the disadvantages is important if we want to understand patients’ digital capabilities, and the inclusion of the next of kin can be evident in unveiling challenges that are unknown and not easily visible or when trying to reach patients with digital challenges through digital means.

Studies by Kayser et al [30] and Kanoe et al [31] indicated that patients’ abilities to interpret and understand digital health–related services and their benefits are important for the successful implementation of eHealth services—an argument that our findings support. Health literacy in both digital and physical contexts is important if we want to understand how to better design and implement services. Our participants’ statements support the argument that communication through digital means cannot be viewed as similar to face-to-face communication and that an emphasis on digital health literacy demonstrates how health systems are demanding different capabilities from the patients [13]. We argue that it is important to communicate the purposes of digital services so that both the patient and their next of kin know why they participate and how it can benefit them. Therefore, it is important to make it as clear as possible that digital health services can benefit the patient and that these services are developed to support information, communication, and dialogue between patients and health professionals. However, our findings suggest that even after interpreting and understanding the purposes of digital health services, some patients may still experience challenges when using digital tools.

Therefore, it is important to understand how and why patients learn digital skills, particularly because both experience with digital devices and estimation of the value of digital tools have been highlighted as key factors for digital practices [5,18]. Our findings indicate that a combination of these factors is important, as recognizing the value of digital tools was not enough to facilitate the necessary learning process for some of our participants. Instead, our participants described the use of digital tools as complex and continuous processes in which automation of skills, assistance from others, and time to relearn forgotten knowledge were necessary and important facilitators for learning and understanding digital tools as well as becoming more comfortable and confident in the use of digital health services. This was particularly important, as it was more encouraging for our participants to learn digital tools when they felt secure, instead of feeling afraid and anxious, a point that Bailey et al [18] also highlighted. The value of digital solutions and the will to learn were greater when challenges were viewed as something to overcome and learn from instead of something that created a feeling of being stupid. This calls for attention on how to simplify and explain digital tools and services so that users do not feel alienated. Our findings also support the argument that digital health literacy should take into account emotional well-being related to digital practice [32].

The various perspectives that our participants provided regarding the use of digital tools in the health care system indicate that patients are affected by the use of digital health services and their own capabilities to use digital tools. Murray et al [33] argued that the use of digital tools in health sectors has the potential to improve health and health delivery by improving efficacy, efficiency, accessibility, safety, and personalization, and our participants also highlighted these positive aspects. However, different studies found that some patients, particularly older adults considered socially vulnerable, have lower digital health literacy [10,34,35], which is an important determinant of health and may widen disparities and inequity in health care [16]. Studies on older adult populations’ adaptation to information and communication technology show that engaging with this technology can be limited by the usability of technology, feelings of anxiety and concern, self-perception of technology use, and the need for assistance and inclusive design [36]. Our participants’ experiences with digital practices support the importance of these focus areas, especially when primarily older patients are admitted to hospitals. Furthermore, our findings indicate that some older patients who used to view themselves as being engaged in their own health care felt more distanced from the health care system because of digital services, and some who did not have the capabilities to use digital tools felt that they were treated differently compared to the rest of society. They did not necessarily view themselves as vulnerable but felt vulnerable in the specific experience of trying to use digital services because they wished that they were more capable. Moreover, this was the case for patients with physical and cognitive difficulties, as they were not necessarily aware of the challenges before experiencing them. Drawing on the phenomenological and feministic approach by Ahmed [37], these challenges that make patients feel vulnerable are not necessarily visible to others but can instead be viewed as invisible institutional “walls” that do not present themselves before the patient runs into them. Some participants had to experience how their physical, cognitive, or communicative difficulties affected their digital practice to realize that they were not as digitally capable as they once were or as others in society. Furthermore, viewed from this perspective, our findings could be used to argue that digital capabilities should be viewed as a privilege tied to users’ physical bodies and that digital services in the health care system are indirectly making patients without this privilege vulnerable. This calls for more attention to the inequities that digital tools and services create in health care systems and awareness that those who do not use digital tools are not necessarily indifferent about the consequences. Particularly, in a context such as the Danish one, in which the digital strategy is to create an intertwined and user-friendly public digital sector for everyone, it needs to be understood that patients have different digital capabilities and needs. Although some have not yet had a challenging experience that made them feel vulnerable, others are very aware that they receive different treatment and feel that they are on their own or that the rest of the society does not care about them. Inequities in digital health care, such as these, can and should be mitigated or prevented, and our investigation into the experiences with digital practices can help to show that we are creating standards and infrastructures that deliberately exclude the perspectives of those who are most in need of the services offered by the digital health care system [8]. Therefore, our findings support the notions that flexibility is important in the implementation of universal public digital services [17]; that it is important to adjust systems in accordance with patients’ eHealth literacy and not only improve the capabilities of individuals [38]; and that the development and improvement of digital health literacy are not solely an individual responsibility but are also tied to ways in which institutions organize, design, and implement digital tools and services [39].

### Limitations

This qualitative study provided novel insights into the experiences with public digital health services from the perspective of patients in the Danish context, enabling a deeper understanding of how digital health services and digital tools are experienced and used. This helps build a solid foundation for future interventions aimed at digital health literacy and digital health interventions. However, this study has some limitations. First, the study was conducted in a country where digitalization is progressing quickly, and people, therefore, are accustomed to this pace. Therefore, readers must be aware of this. Second, the study included patients with different neurological conditions; some of their digital challenges were caused or worsened by these neurological conditions and are, therefore, not applicable to all patients in the health system. However, the findings provided insights into the patients’ digital practices before their conditions and other challenges not connected to neurological conditions shared by patients. Third, the study was broad, and although a large number of informants was included, from a qualitative research perspective, we would recommend additional research in this field to develop interventions that target digital health literacy and the use of digital health services.

### Conclusions

Experiences with digital tools and digital health services are complex and multifaceted. The advantages in communication, finding information, or navigating through one’s own health course work as facilitators for engaging with digital tools and digital health services. However, this is not enough on its own. Furthermore, feeling secure and motivated and having time to relearn and practice skills are important facilitators. Engagement in digital practices for the examined population requires access to continuous assistance from their social network. If patients do not meet requirements, digital health services can be experienced as exclusionary and a source of concern. Physical, cognitive, and communicative difficulties might make it impossible to use digital tools or create more challenges that require assistance. Digitalization of the health care system means that patients do not have the choice to opt out of using digital services without having consequences, resulting in them receiving a different treatment than others. To ensure digitalization does not create inequities in health, it is necessary for developers and the health institutions that create, design, and implement digital services to be aware of differences in digital health literacy and to focus on simplifying communication with patients and next of kin through and about digital services. It is important to focus on helping individuals meet the necessary conditions and finding flexible solutions for those who do not have the same privileges as others if the public digital sector is to work for everyone.

## Abbreviations

COREQ: Consolidated Criteria for Reporting Qualitative Research
